# Artificial intelligence and multimodal imaging in orthopaedics: from technological advances to clinical translation

**DOI:** 10.3389/fmed.2025.1728248

**Published:** 2026-01-09

**Authors:** Guangan Luo, Shuanglong Tan, Lincong Luo, Konghe Hu

**Affiliations:** 1 Guangdong Medical University, Zhanjiang, China; 2 Yue Bei People’s Hospital Postdoctoral Innovation Practice Base, Southern Medical University, Guangzhou, China

**Keywords:** multimodal imaging, artificial intelligence, deep learning, orthopaedic diagnosis and treatment, clinical translation

## Abstract

The integration of multimodal medical imaging with artificial intelligence (AI) is potentially catalysing a paradigm shift in orthopaedic diagnosis and treatment, moving beyond experience-based practices toward intelligent, data-driven precision medicine. This narrative review synthesizes recent key evidence across imaging modalities and AI frameworks, and highlights the translational gap that persists between algorithmic development and real-world clinical implementation. By combining complementary information from X-ray, CT, MRI, PET, ultrasound, and biochemical data, multimodal AI overcomes the inherent limitations of single-modality approaches, enabling more comprehensive structural, functional, and metabolic assessments. Recent advances demonstrate broad applications, including accurate fracture detection and classification, differentiation of benign and malignant bone tumours, quantitative assessment of osteoarthritis, risk prediction for osteoporosis, and intelligent preoperative planning and intraoperative navigation. Moreover, multimodal AI facilitates efficacy prediction and personalised treatment decision-making, positioning future systems as AI-assisted decision-support tools that support surgeons in surgical strategy, implant design, and long-term follow-up. Nevertheless, significant challenges remain, particularly in data heterogeneity, model generalisation, interpretability, and clinical integration. Progress in constructing standardised multimodal databases, developing self-supervised and multi-task learning strategies, and strengthening ethical–regulatory frameworks will be essential for clinical translation. Ultimately, multimodal AI holds immense potential to transition from laboratory validation to routine practice, delivering safer, more efficient, and precise diagnostic and therapeutic solutions for orthopaedic patients.

## 1 Introduction

Orthopaedic diseases are characterised by high incidence, disability, and recurrence rates, and thus represent a major global public health concern. These conditions frequently lead to functional impairment and impose substantial healthcare and socioeconomic burdens (1, 2). According to the 2023 Global Burden of Disease study published in the Lancet, nearly 170 million people are affected by orthopaedic diseases worldwide each year (3). Among them, fractures, osteoarthritis, and bone tumours are the most prevalent and high-risk conditions. In China, osteoporotic fractures affect 32.1% of individuals aged 65 years and older, while knee osteoarthritis affects over 15.2% of the same population (4, 5). Moreover, the ageing population, coupled with the rising incidence of sports injuries and bone tumours, is intensifying the demand for orthopaedic diagnosis and treatment, thereby generating increasingly complex clinical challenges.

In recent years, AI has begun to play an increasingly important role in orthopaedic practice. Current applications include assisting fracture identification, grading osteoarthritis severity, supporting tumour assessment, and improving preoperative planning. As highlighted by Khojastehnezhad et al. (6), AI has enhanced diagnostic efficiency and consistency across multiple orthopaedic scenarios, although challenges related to data quality, model transparency, and clinical validation still limit widespread adoption. These developments provide essential context for exploring how multimodal imaging integrated with AI may further advance precision orthopaedic care.

Currently, precise diagnosis of orthopaedic diseases remains difficult. Missed or misdiagnosed fractures may result in long-term disability and high societal costs (7). In addition, bone and soft tissue tumours encompass diverse subtypes, and differentiating benign from malignant forms continues to rely heavily on radiologists’ expertise (7). Early-stage osteoarthritis often lacks specific symptoms and reliable diagnostic markers, making timely identification particularly difficult (5). Although DXA-based bone density measurement is the gold standard for osteoporosis assessment, fracture risk is also influenced by factors such as trabecular microarchitecture and bone metabolism (8). Consequently, diagnostic strategies relying on a single modality are increasingly inadequate to meet the requirements of precision treatment.

Medical imaging plays a pivotal role in orthopaedic evaluation, with each modality providing distinct advantages and applications. Specifically, X-rays are simple, rapid, and cost-effective, making them well suited for initial screening and follow-up (9). CT offers high spatial resolution for delineating bony structures and is widely employed in assessing complex fractures and in preoperative 3D reconstruction (10). MRI, with superior sensitivity to soft tissue, is particularly valuable for assessing cartilage, ligaments, and tumour invasion (11). Ultrasound is safe, real-time, and well suited for detecting superficial joint synovial lesions (12). PET/CT integrates anatomical and metabolic information, thereby providing unique value in assessing tumour activity and metastasis (13). DXA remains the standard for diagnosing osteoporosis; however, it measures only bone mass and fails to reflect bone quality or structural heterogeneity (14). Nevertheless, reliance on a single modality cannot comprehensively capture the structural, biochemical, and functional features of complex bone diseases, thereby restricting its utility in precision medicine.

In parallel, the rapid advancement of AI, particularly deep learning, has consequently driven a disruptive transformation in medical image analysis (15). In recent years, applications of AI in orthopaedics have expanded markedly. These applications encompass lesion detection, tissue segmentation, disease classification, and preoperative risk assessment. Moreover, integrating AI has enhanced the efficiency and consistency of image interpretation; however, evidence for direct improvements in patient outcomes remains limited and is largely inferential (16, 17). However, most current AI models are constrained by reliance on single-modality data, leading to redundancy and limited generalisability. For example, fracture detection models trained on X-rays perform poorly on complex 3D anatomy, while MRI-based soft tissue segmentation models cannot be directly transferred to CT environments (18). Where applicable, we explicitly distinguish research-stage multimodal models from FDA/CE-cleared tools and note that evidence for improved patient outcomes remains limited.

To overcome these limitations, a novel fusion strategy combining multimodal imaging with AI has emerged. By integrating heterogeneous imaging information from CT, MRI, PET, and other modalities at the data, feature, or decision levels, AI systems emulate clinicians’ ability to comprehensively interpret multimodal images and clinical data. This approach holds strong potential to enhance disease recognition, predict risk stratification, and guide personalised treatment (19). Notably, multimodal AI has already been applied in clinical contexts, including fracture classification, benign–malignant tumour differentiation, joint degeneration grading, osteoporosis risk prediction, intraoperative navigation, and postoperative complication assessment. These applications have demonstrated promising adaptability and considerable clinical potential (20, 21). At the same time, challenges including cross-modal coordination, model interpretability, generalisation, and clinical feasibility are attracting growing attention (22).

**Figure 1:**
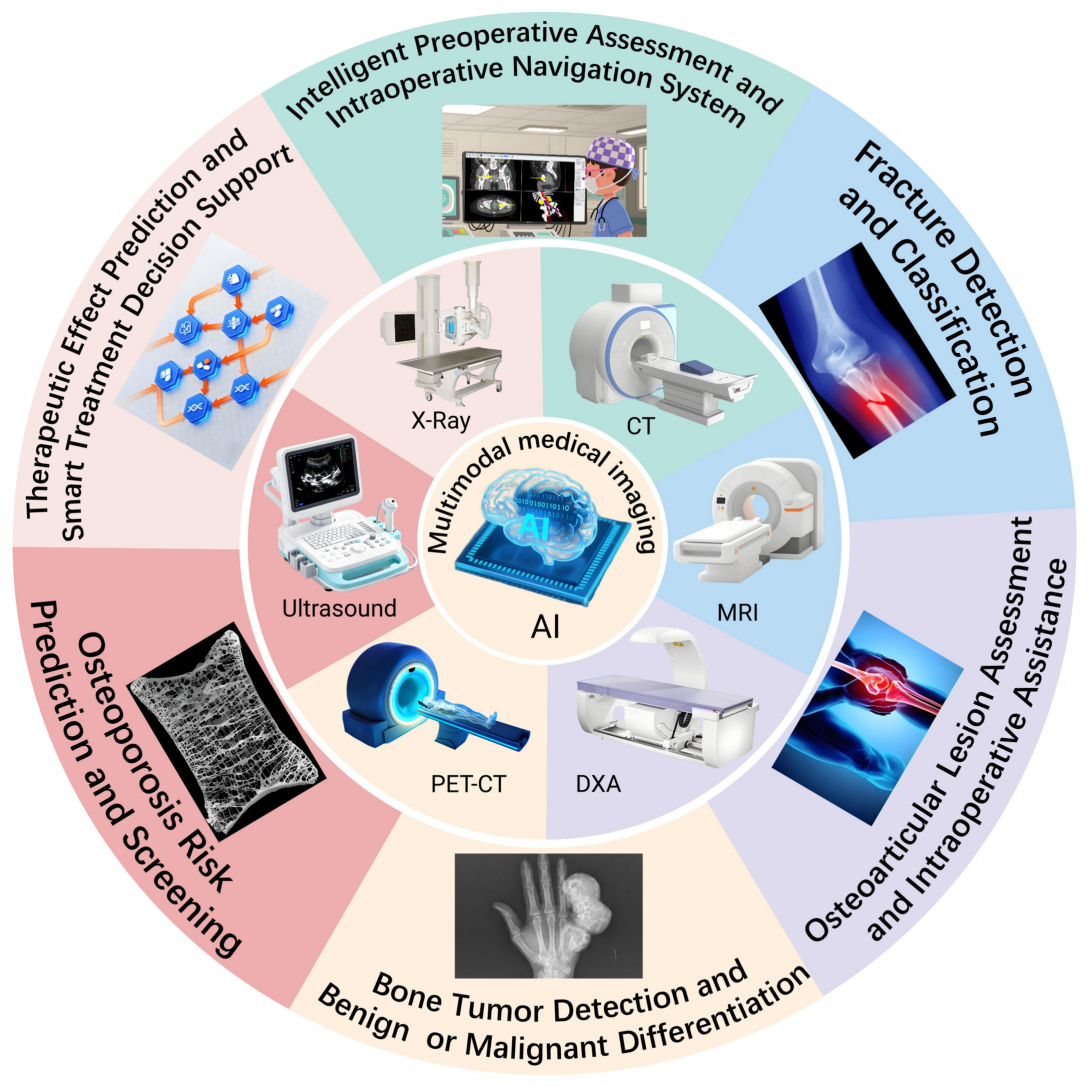
Schematic representation of multimodal AI integration in orthopaedic diagnosis and treatment, highlighting the role of AI in analyzing imaging data and assisting in decision-making.

Hence, this review systematically evaluates advancements in multimodal imaging integrated with AI technologies for orthopaedic disease diagnosis and therapeutic interventions. It focuses on core technical frameworks and integration strategies, with particular emphasis on practical applications such as fractures, osteoarthritis, bone tumours, osteoporosis, and intraoperative navigation. The evaluation further considers research quality, clinical translation value, and key challenges. Furthermore, it explores future directions, including model generalisation, fairness, interpretability, and the potential of multimodal AI in advancing smart orthopaedics and precision medicine. Ultimately, this review seeks to provide theoretical insights and practical guidance for clinical applications, thereby promoting the high-quality development and standardised implementation of AI in orthopaedic imaging (Figure 1).

## 2 Literature search and selection

A focused literature search was conducted using the PubMed, Scopus, and Web of Science databases for studies published between 2015 and 2025. Search terms included combinations of “multimodal imaging,” “artificial intelligence,” “orthopaedics,” “deep learning,” “X-ray,” “CT,” and “MRI.” Studies were included if they: (1) reported applications of AI to musculoskeletal imaging or orthopaedic clinical workflows; (2) involved human subjects or clinically relevant imaging datasets; and (3) were published in English. Exclusion criteria included: (1) studies unrelated to orthopaedic conditions; (2) papers without accessible full text; and (3) purely technical reports lacking clinical relevance.

## 3 Multimodal imaging and artificial intelligence technologies

### 3.1 Types of multimodal imaging

Multimodal medical imaging integrates data from X-ray, CT, MRI, PET, SPECT, and ultrasound, thereby enabling a comprehensive structural, functional, and metabolic assessment of orthopaedic diseases (23) (Table 1). Each modality provides complementary strengths—ranging from high spatial resolution and advanced tissue discrimination to rapid acquisition and functional imaging—thereby extending diagnostic capability. For instance, X-rays and CT delineate bone structures, MRI excels in detecting marrow lesions and soft-tissue pathology, while PET highlights metabolic activity in bone tumours (24). In clinical practice, registration and fusion align multimodal data for simultaneous structural and functional visualisation, which in turn improves diagnostic accuracy and streamlines surgical planning (25). Notably, CT–MRI fusion is validated for complex spinal navigation (26), whereas PET–CT remains indispensable for tumour staging and metastasis evaluation (11).

**Table 1:** Comparative performance of imaging modalities in orthopaedics.

Imaging modality	Spatial resolution	Soft tissue contrast	Cost & accessibility	Scan duration	Radiation dose	Clinical advantages	Major limitations
X-ray	High (0.5–1.0 mm)	Low	Very low; widely available at primary care level	~few minutes	Low	First-line for fracture diagnosis; fast and cost-effective	2D projection only; poor soft tissue contrast
CT	Very high (~0.5 mm)	Moderate	Moderate; widely available	~10 min	Moderate	Detailed anatomical structures; essential for complex fractures and preoperative planning	Limited soft tissue contrast; higher radiation than X-ray
MRI	Moderate (~1–2 mm)	Very high	High; mainly tertiary hospitals	30–60 min	None	Excellent soft tissue delineation; evaluation of tumour infiltration and cartilage	High cost; long scan time; lower spatial resolution than CT
Ultrasound	Moderate (frequency-dependent)	Moderate	Low; bedside accessible	~30 min	None	Real-time dynamic assessment; no radiation; suitable for superficial lesions and joint effusion	Operator-dependent; cannot assess intraosseous structures
PET/CT	Low (PET ~4–6 mm)	Very high (functional/metabolic)	Very high; limited to specialised centres	90–120 min	High	Sensitive for tumour staging and bone metastasis detection	Low resolution; high cost; significant radiation exposure
DXA	Moderate	Low	Low; widely available	~5–10 min	Very low	Quantitative bone mineral density; gold standard for osteoporosis diagnosis	Cannot assess bone quality or microarchitecture; limited predictive value for fracture risk

MRI provides superior soft tissue contrast compared with CT, whereas CT achieves higher spatial resolution. PET primarily reflects metabolic activity and requires CT for anatomical localisation. DXA quantifies bone mineral density but does not capture structural or qualitative aspects.

### 3.2 Alignment and fusion of multimodal images

Robust analysis of multimodal images critically depends on high-precision registration and fusion algorithms. The core objectives are precise spatial alignment (registration) and semantic-level integration (fusion) across modalities. Mutual-information approaches are widely adopted for heterogeneous data (e.g., MRI–CT, PET–CT) and generally robust; however, their performance declines under distortion or poor image quality (27). Moreover, deep-learning registration, particularly unsupervised U-Net or Transformer variants, substantially enhances processing speed and non-rigid accuracy (28, 29). For example, VoxelMorph achieves high-dimensional registration within seconds and is validated for rapid alignment of preoperative spinal CT with intraoperative cone-beam images (30). Because bone–soft-tissue boundaries are indistinct, registration requires boundary-sensitive optimisation; accordingly, attention mechanisms and multi-scale pyramids have become active research directions (31). Fusion strategies have evolved from pixel-level to feature-level and, more recently, to decision-level approaches. While pixel-level fusion enhances detail, it remains sensitive to modality mismatch. By contrast, feature-level fusion preserves complementary information, thereby improving downstream lesion detection. Optimising fusion to balance structural fidelity with semantic compensation is critical for clinically significant tasks such as cartilage-degeneration analysis and tumour-boundary detection.

### 3.3 Core algorithms in orthopaedic AI

Orthopaedic image analysis relies heavily on deep learning, with convolutional neural networks (CNNs) serving as the mainstay for image recognition (32). Core tasks include lesion detection, anatomical segmentation, disease grading, and decision support before and during surgery. More recently, Vision Transformers have gained momentum due to their superior capacity for global modelling. In fracture detection, YOLO and Faster R-CNN enable rapid and accurate localisation (33, 34). Similarly, U-Net and its variants excel in bone-tumour segmentation, particularly in accuracy and boundary preservation (35). In osteoarthritis grading, multimodal models that integrate imaging with clinical indicators demonstrate promising predictive power (36). Additionally, generative adversarial networks have been increasingly applied to denoising, super-resolution, and few-shot learning in orthopaedic imaging (37).

### 3.4 Construction of multimodal AI systems

**Figure 2:**
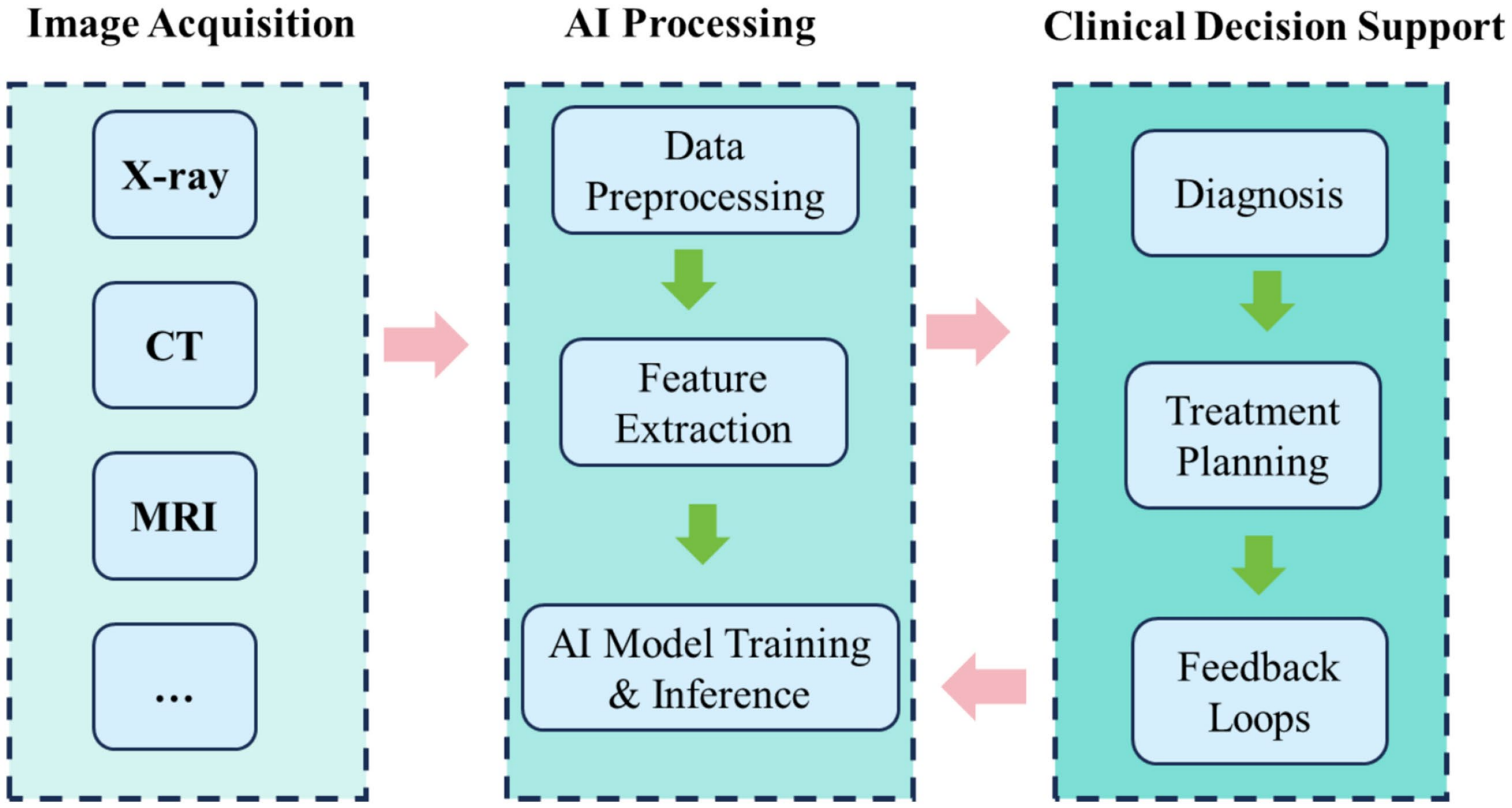
Workflow of multimodal imaging integration with AI in clinical practice.

Constructing clinically transferable multimodal AI systems generally involves five stages: data acquisition and preprocessing, feature extraction and alignment, model construction and training, evaluation and validation, and clinical integration. During preprocessing, images from diverse modalities must be normalised, enhanced, and registered to ensure consistency of inputs. Feature extraction must capture complementary spatial resolution and anatomical features across modalities. During training, multi-task learning and attention mechanisms are often employed to improve generalisation. For example, dual-channel networks fusing MRI soft-tissue features with CT bone structures enable structure-aware models through fusion layers (38). Ultimately, final systems require multicentre validation and seamless integration into clinical platforms such as PACS and intraoperative navigation (Figure 2).

## 4 Applications of multimodal AI in orthopaedic diseases

This section systematically reviews advances in multimodal imaging AI across the entire clinical continuum. Specifically, the scope encompasses disease detection, risk assessment, treatment decision-making, and intraoperative support. The emphasis is placed on core orthopaedic clinical tasks. This section also examines why multimodal fusion outperforms single-modality approaches in diagnostic performance, predictive accuracy, and clinical utility. Each subsection highlights representative clinical scenarios, summarises model strategies, validation methods, and practical applications, and further identifies translational bottlenecks and future directions.

### 4.1 Fracture detection and classification

**Figure 3:**
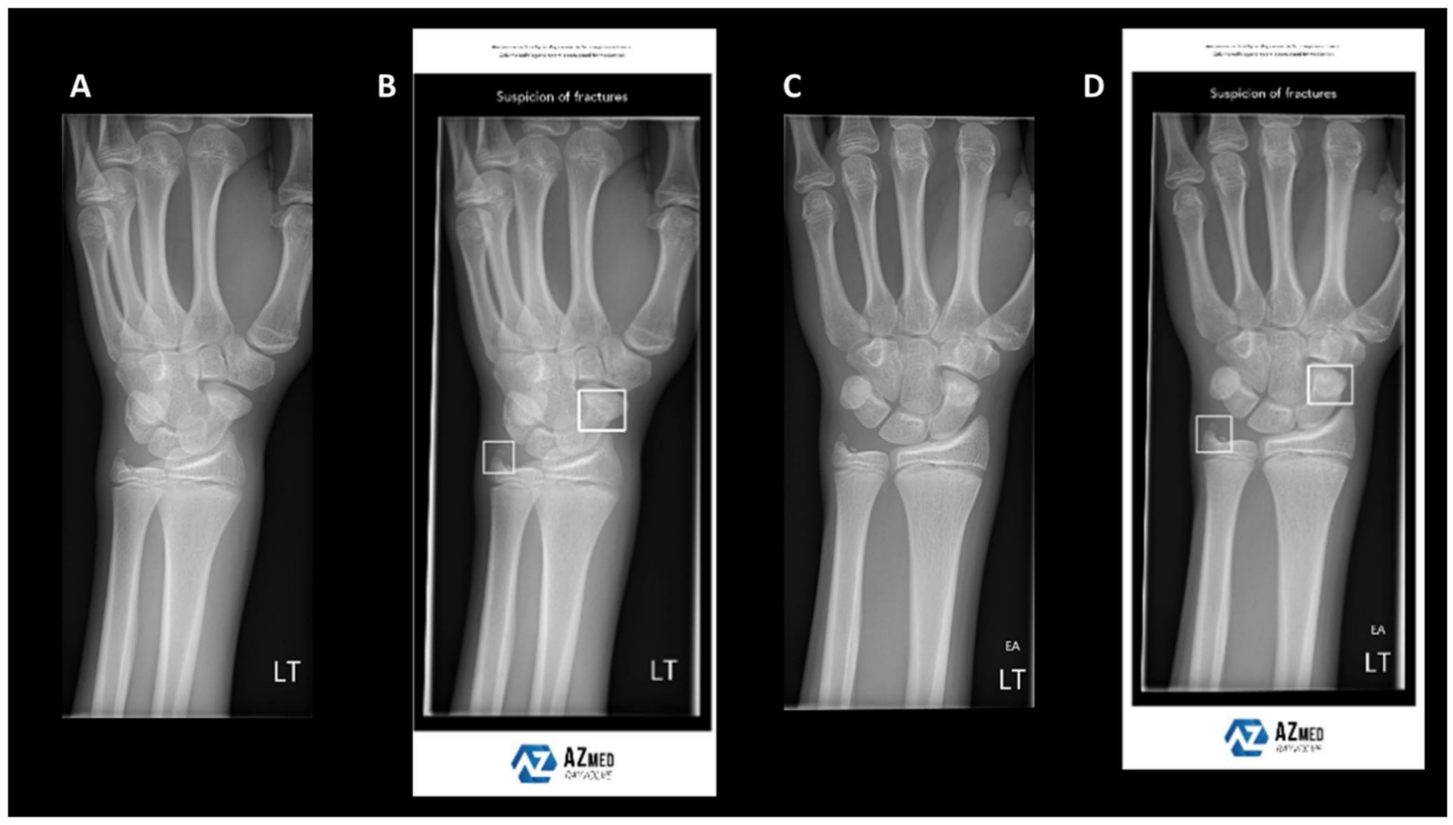
Example wrist radiographs illustrating AI-assisted fracture detection. **(A,C)** Standard radiographs; **(B,D)** radiographs with AI-based highlighting of suspected fracture regions. Reproduced under terms of the CC-BY license (39). Copyright 2024, BJR.

Fracture detection and classification represent some of the earliest and most extensively adopted AI applications in orthopaedics. In routine practice, emergency cases are screened using X-rays, whereas complex fractures require CT for detailed anatomical information. However, single-modality imaging has intrinsic limitations, often resulting in missed or misclassified fractures, particularly subtle or complex patterns. Recently, deep-learning models trained on X-rays have been increasingly deployed for fracture recognition. Although these models perform well in selected scenarios, their accuracy declines in the presence of structural overlap or poor image quality (39) (Figure 3). To address these limitations, researchers have fused CT and X-ray data, thereby improving sensitivity and specificity in fracture detection. For example, the FDA-cleared BoneView system automatically annotates suspected fractures on X-rays and cross-validates with CT findings, thereby enhancing detection rates and reporting efficiency in emergency settings. Furthermore, Liu et al. (40) integrated X-rays with demographic and medical history data to predict dissatisfaction after total knee arthroplasty, achieving AUCs of 0.832–0.891 and markedly outperforming models based solely on clinical data. These findings underscore the critical prognostic value of bone-imaging features.

In the diagnosis of spinal fractures, multimodal strategies likewise demonstrate superior performance. Geng et al. (41) extracted 1,197 radiomics features from multi-sequence MRI (T1/T2) and used an XGBoost classifier to distinguish osteoporotic from metastatic vertebral compression fractures, reaching an external-validation AUC of 0.905; adding clinical features increased AUC to 0.982. Although developed for specific fracture types, the fusion methodology is broadly applicable to general fracture classification. Beyond diagnosis, multimodal AI is progressively expanding into fracture subclassification and treatment decision support. Hip fractures exemplify this trend: X-rays support preliminary classification, whereas CT provides quantitative assessment of displacement and fragment distribution. Fusion AI systems can then estimate the likelihood of successful screw fixation or the need for arthroplasty (42). Several studies also report personalised AI assistants that integrate patient X-rays with basic clinical data to recommend surgical plans, thereby supporting physician decision-making (43).

Notably, some studies extend “multimodal” to multi-view fusion, in which anteroposterior and lateral X-rays serve as complementary inputs within a single modality to enhance performance. In a meta-analysis of seven commercial systems, Husarek et al. found that dual-view inputs significantly increased sensitivity and specificity for long-bone fractures (44). Moreover, collaborative AI–physician interpretation further improves diagnostic accuracy (45). This “generalised multimodal” strategy demonstrates strong feasibility for real-world deployment.

In summary, multimodal AI provides three major advantages in fracture detection and detailed classification: (1) Diagnostic—expanding from single X-ray to multimodal inputs (X-ray + CT/MRI) improves recognition of complex and occult fractures; (2) Predictive—integrating imaging with clinical information increases the accuracy of predicting surgical outcomes and patient responses; and (3) Translational—a small number of AI systems—mostly limited to single-modality or task-specific applications—have obtained FDA/CE clearance, whereas multimodal fusion systems remain primarily at the research or pilot-evaluation stage. Looking forward, advances in data quality and optimisation of multimodal algorithms are expected to enable deeper AI integration across all stages of fracture care.

### 4.2 Evaluation of bone and joint lesions and intraoperative

Osteoarthritis (OA) is the most common chronic degenerative joint disease, and radiographic assessment continues to play a central role in both diagnosis and monitoring of disease progression. However, conventional radiographic evaluation is inherently subjective and remains heavily dependent on reader expertise. Consequently, these methods often fail to capture early changes, including cartilage degeneration, synovial inflammation, and osteophyte formation (46). Recently, AI, particularly multimodal image fusion, has enabled objective quantitative diagnosis, early prediction, and classification-driven interventions for OA.

**Figure 4:**
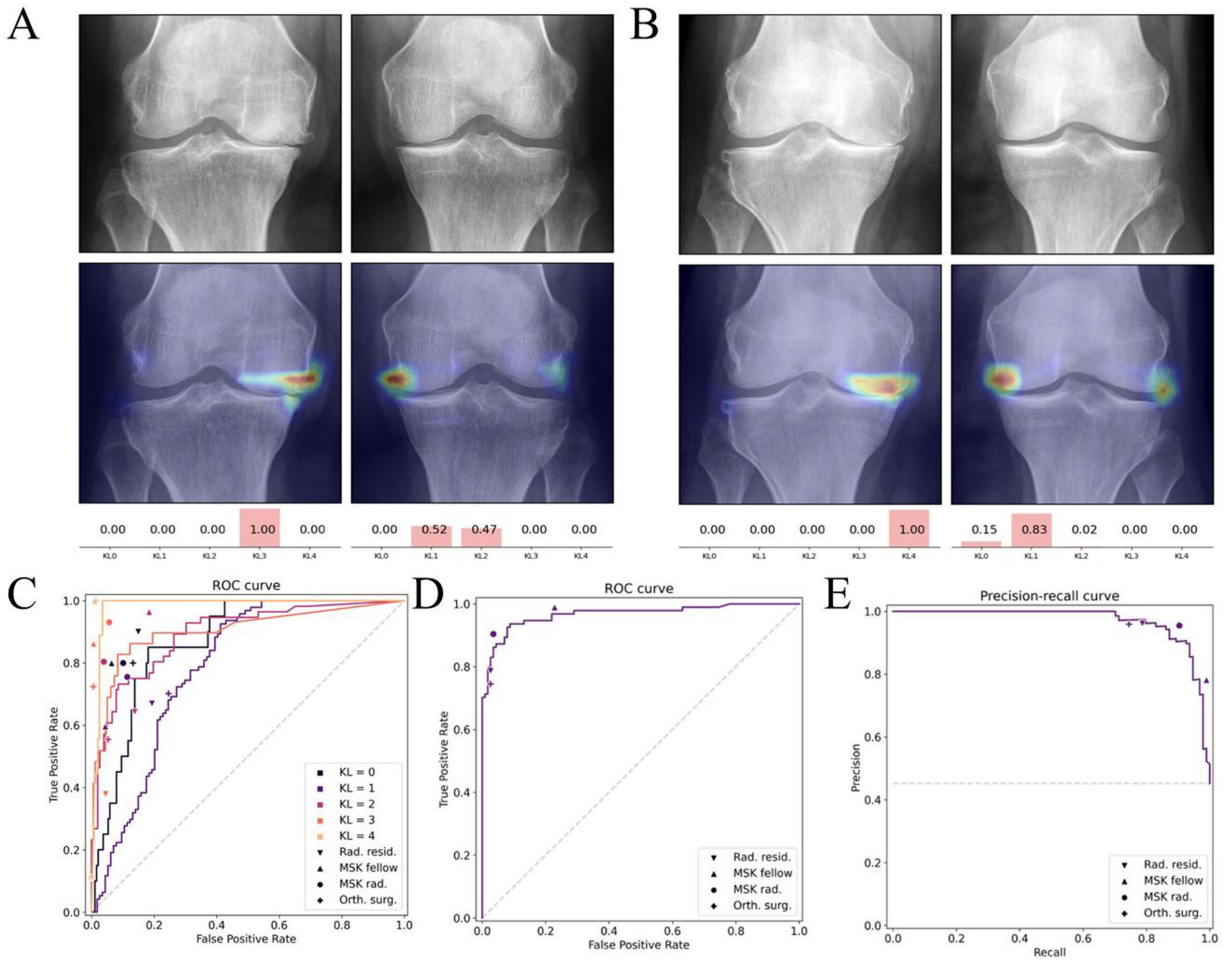
Deep learning–based Kellgren–Lawrence (KL) grading of knee osteoarthritis. **(A,B)** Example radiographs with corresponding model attention heatmaps and predicted KL probabilities. **(C–E)** Diagnostic performance of the deep learning model compared with human readers, illustrated by receiver operating characteristic (ROC) and precision–recall curves. Reproduced under terms of the CC-BY-NC-ND license (47). Copyright 2025, Osteoarthritis and Cartilage.

For early diagnosis, AI models automatically detect features such as joint-space narrowing, osteophyte formation, and contour changes by analysing X-rays, MRI, and clinical data. As a result, this approach significantly enhances diagnostic consistency. Notably, deep learning–based automated Kellgren–Lawrence (KL) grading models achieve an AUC of 0.92 in external validation, surpassing the diagnostic consistency of most primary radiologists (47) (Figure 4). Moreover, MRI remains pivotal for early OA assessment owing to its sensitivity to soft tissues including cartilage, synovium, and bone marrow lesions. For example, Khan et al. (48) developed a multimodal CNN that integrates T1/T2-weighted MRI with serum inflammatory markers, thereby enabling personalised prediction in high-risk OA populations.

In disease staging and progression monitoring, multimodal AI systems integrating structural and functional imaging (e.g., quantitative MRI, PET–MRI) represent a major advance. These systems track temporal changes in cartilage volume, T2-mapping signals, and bone marrow oedema, thereby allowing prediction of disease progression trajectories (49). Lee et al. (50) developed an OA progression model that integrates MRI with clinical data (e.g., gait velocity), predicting KL grade changes over 4–5 years with an AUC of 0.79–0.82. This model further demonstrated strong temporal stability and generalisability. By contrast, this dynamic approach outperforms static KL grading and facilitates timely identification of rapidly progressing patients.

Furthermore, AI is increasingly applied to personalised treatment decision support in OA. Several models integrate age, BMI, activity, joint alignment, and radiographic severity to predict the efficacy of non-surgical interventions (e.g., intra-articular injections, rehabilitation) or determine the need for knee replacement. Chen et al. (51) reported that multimodal AI–guided treatment achieved significantly greater one-year functional improvement than experience-based decision-making (17.6% improvement, *p* < 0.05). Importantly, AI applications in OA are now showing potential for future clinical applicability, although most systems remain at the research stage. For instance, the Stanford OA AI platform integrates automated MRI segmentation with OA risk scoring, thereby enabling automated structural assessment. Moreover, several products have already gained FDA approval for OA progression monitoring and preoperative intervention assessment (52, 53). Collectively, these developments highlight multimodal AI as a critical tool for long-term OA management and precise staging.

In summary, multimodal AI has shifted OA care from “static image-assisted judgment” to “dynamic progression prediction,” and ultimately to “personalised intervention planning.” This evolution enhances early detection and provides strong support for establishing personalised and proactive management models for OA.

### 4.3 Bone tumor detection and benign/malignant diagnosis

Bone tumours, with their low incidence, heterogeneous subtypes, and diverse imaging features, have traditionally depended on the subjective interpretation of senior radiologists (54). Although X-ray, CT, and MRI are essential for localisation and morphological assessment, they remain inadequate for differentiating benign from malignant tumours, grading lesions, and delineating margins (55). Recently, advances in AI-driven multimodal imaging have created new opportunities for early detection and precise characterisation of bone tumours.

At the screening and localisation stage, AI models trained on large-scale bone tumour X-rays or CT scans can rapidly detect and automatically annotate suspected lesions. Numerous studies have applied CNNs to long-bone X-rays, achieving detection sensitivities above 90% (56). AI-based bone scan tools facilitate the detection of multiple bone metastases, thereby reducing misdiagnosis and missed diagnoses. These tools are particularly valuable for patients at high risk of metastasis, such as those with breast or prostate cancer (57).

**Figure 5:**
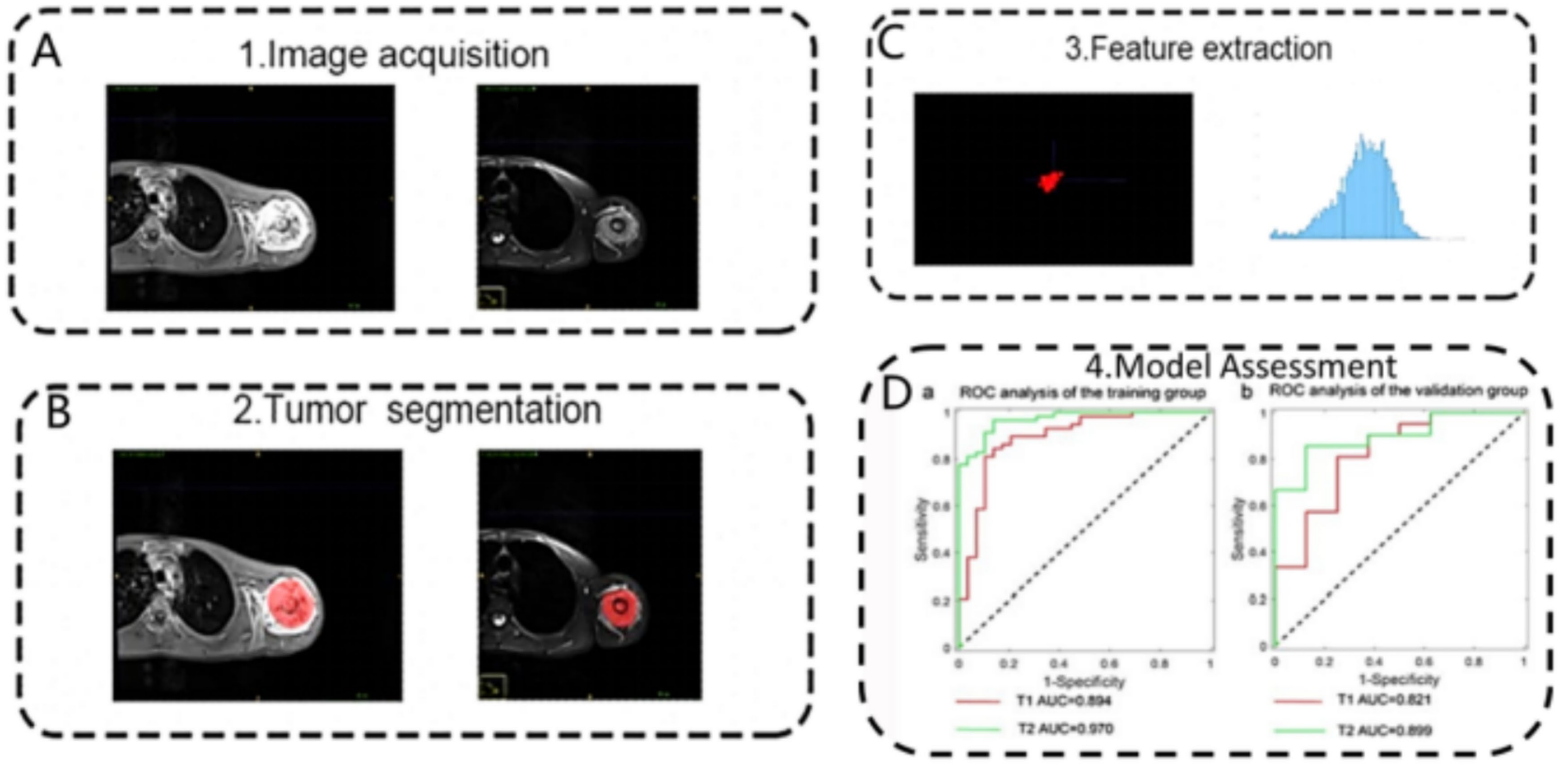
Schematic representation of the radiomics analysis pipeline for differentiating osteosarcoma and chondrosarcoma based on MRI. The process involves: **(A)** image acquisition, **(B)** tumour segmentation, **(C)** feature extraction, and **(D)** model assessment using receiver operating characteristic analysis in training and validation cohorts. Reproduced under terms of the CC-BY-NC-ND license (60). Copyright 2024, Scientific Reports.

To differentiate benign from malignant lesions and determine tumour grade, multimodal AI models achieve superior accuracy. Compared with single-modality analysis, combined MRI–CT evaluation captures structural, signal, and margin variations, thereby markedly enhancing classification performance (58). Zhou et al. (59) designed a three-channel deep learning model that integrates T1-weighted MRI, T2-weighted MRI, and contrast-enhanced CT, achieving an AUC of 0.94 for benign–malignant classification and substantially outperforming single-modality models. Furthermore, radiomics approaches integrated with AI extract hundreds of latent features (e.g., texture, morphology, grey-scale distribution) and combine them with clinical variables (e.g., age, lesion site). Gao et al. (60) established an MRI radiomics model that distinguished osteosarcoma from chondroma with >92% accuracy, offering auxiliary diagnostic support in cases with ambiguous benign–malignant boundaries (Figure 5).

Moreover, AI is becoming increasingly important for histopathological pre-diagnosis and treatment planning. Several studies have explored the prediction of tumour molecular subtypes (e.g., IDH mutation), malignancy grade, and therapy sensitivity using imaging features, thereby providing critical data support for precision medicine (61). Multimodal AI systems now integrate PET–CT functional imaging into workflows, enabling simultaneous analysis of metabolic activity and structural features to optimise biopsy targeting and predict treatment efficacy (62). In addition, AI is accelerating the clinical translation of bone tumour imaging. For example, OsteoDetect provides real-time marking of potential lesions on X-rays, thereby alerting clinicians to suspicious areas (44). Furthermore, high-risk scoring models have been embedded into PACS systems, automatically generating diagnostic recommendations to enhance efficiency and consistency (63). Importantly, these tools are not intended to replace physicians but to strengthen human–AI collaboration by providing structured, quantifiable data support.

In summary, AI-driven bone tumour imaging is rapidly evolving from lesion identification to benign–malignant classification, molecular prognosis prediction, and precise intervention planning. The integration of multimodal AI not only improves diagnostic reliability for complex lesions but also provides a robust foundation for developing personalised treatment strategies. Ultimately, this advancement is poised to usher bone tumour diagnosis and treatment into a new era of intelligent and precise medicine.

### 4.4 Osteoporosis risk prediction and screening

Osteoporosis is a systemic metabolic bone disease marked by reduced bone mass and deterioration of bone microarchitecture. This condition poses a major threat to both quality of life and physical safety in elderly populations. Although DXA remains the gold standard, it has clear limitations, including lengthy testing cycles and low sensitivity for subclinical disease (64). Recently, AI, particularly multimodal approaches, has driven breakthroughs in early identification, risk prediction, and personalised management of osteoporosis.

In bone mineral density (BMD) assessment, integrating multimodal AI with imaging and clinical data has markedly improved predictive accuracy. Notably, several studies have demonstrated “imaging-based BMD estimation” without the need for additional radiation exposure. This strategy extracts trabecular texture features from conventional CT images of the lumbar spine and femur and combines them with machine learning models (65). For example, Wang et al. (66) developed an AI model integrating age, sex, BMI, and CT texture features to predict T-scores < −2.5, achieving an AUC of 0.943. This model outperformed those relying solely on DXA, underscoring its potential as a clinical substitute.

**Figure 6:**
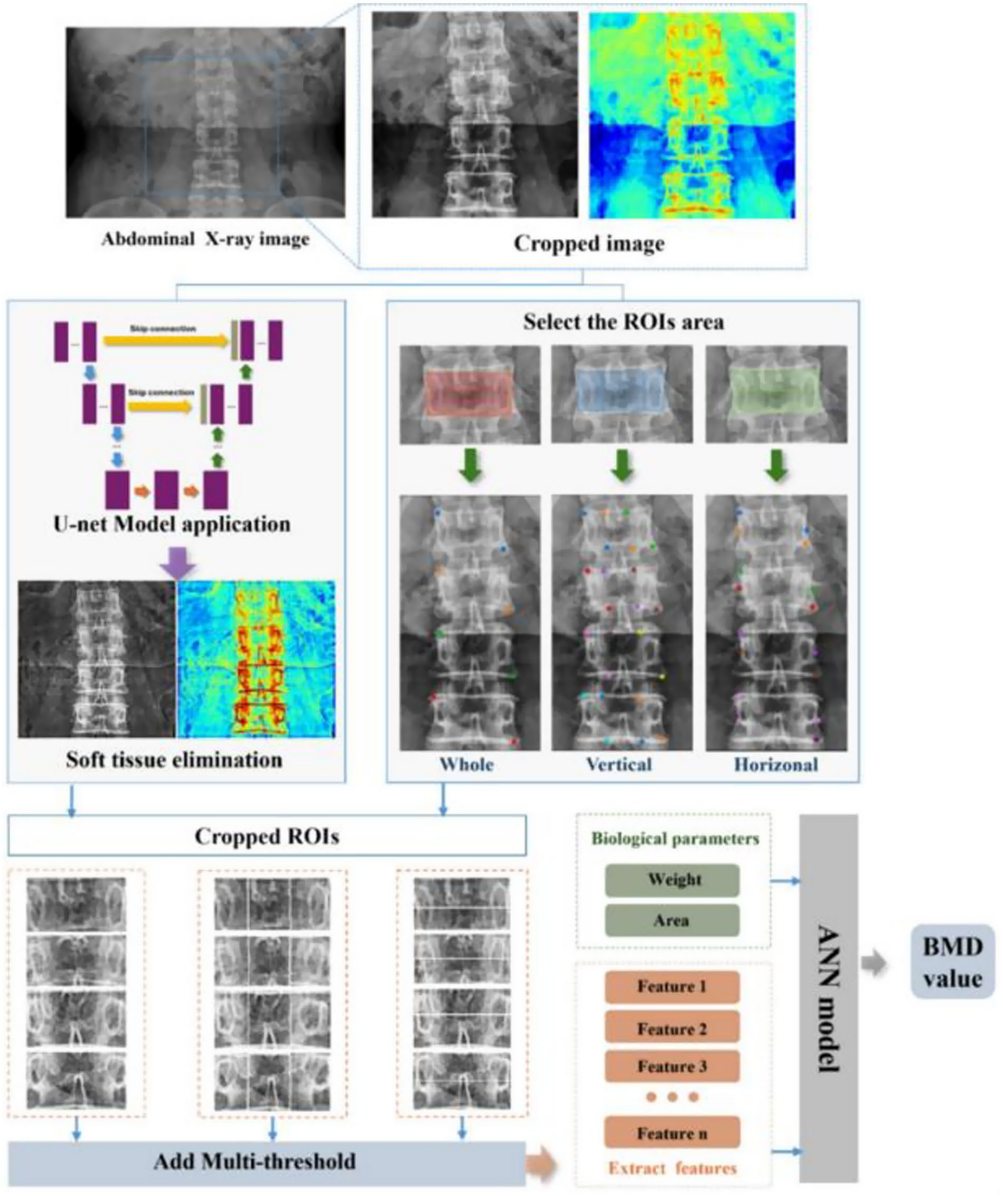
Overall workflow of the proposed U-Net-based deep learning hybrid model for predicting bone mineral density (BMD) from abdominal X-ray images. The process involves image preprocessing using U-Net for soft tissue elimination, region-of-interest (ROI) selection, multi-threshold feature extraction, and integration of biological parameters into an artificial neural network (ANN) to output predicted BMD values. Reproduced under terms of the CC-BY license (71). Copyright 2025, Bioengineering.

Moreover, multimodal AI has also been widely applied to prospective fracture risk prediction. By integrating multidimensional data—including DXA, X-rays, medical history, exercise habits, and biochemical markers—AI identifies high-risk individuals often missed by traditional tools such as FRAX (67, 68). In particular, Kong et al. (69) developed a deep learning network combining spinal X-rays with fracture history, predicting fragility fracture risk within three years with sensitivity above 90%. These models are especially valuable for identifying individuals with “normal BMD but high fragility,” representing a hidden osteoporotic population. Furthermore, advances in AI are enabling personalised management of osteoporosis. By continuously monitoring medication adherence, BMD trajectories, and metabolic indicators, AI models can predict drug response and fracture recurrence risk, thereby enabling dynamic treatment adjustment (70). Zhou et al. (71) demonstrated that a temporal AI model trained on multimodal data predicted one-year BMD trends after six months of medication, achieving accuracy above 85% (Figure 6). Looking ahead, integrating wearable devices and home imaging could make AI-enabled dynamic bone health management more accessible.

In summary, multimodal AI is transforming osteoporosis management from static detection to dynamic prediction and from population-level screening to individualised care. Ultimately, this advancement enhances risk identification and management precision, providing technological support for bone health strategies in ageing societies.

### 4.5 Intelligent preoperative assessment and intraoperative navigation systems

Preoperative planning and intraoperative navigation are indispensable for precision surgery, particularly in complex orthopaedic procedures, as they improve safety and reduce postoperative complications. Conventional methods, which rely on two-dimensional imaging and surgeon experience, restrict intuitive three-dimensional reconstruction and real-time adaptation to intraoperative changes (72). Recently, the emergence of multimodal AI has driven a paradigm shift in orthopaedic surgery. The integration of CT, MRI, preoperative X-rays, 3D modelling, and real-time intraoperative imaging has enabled intelligent navigation systems defined by precision, adaptability, and personalisation (73).

**Figure 7:**
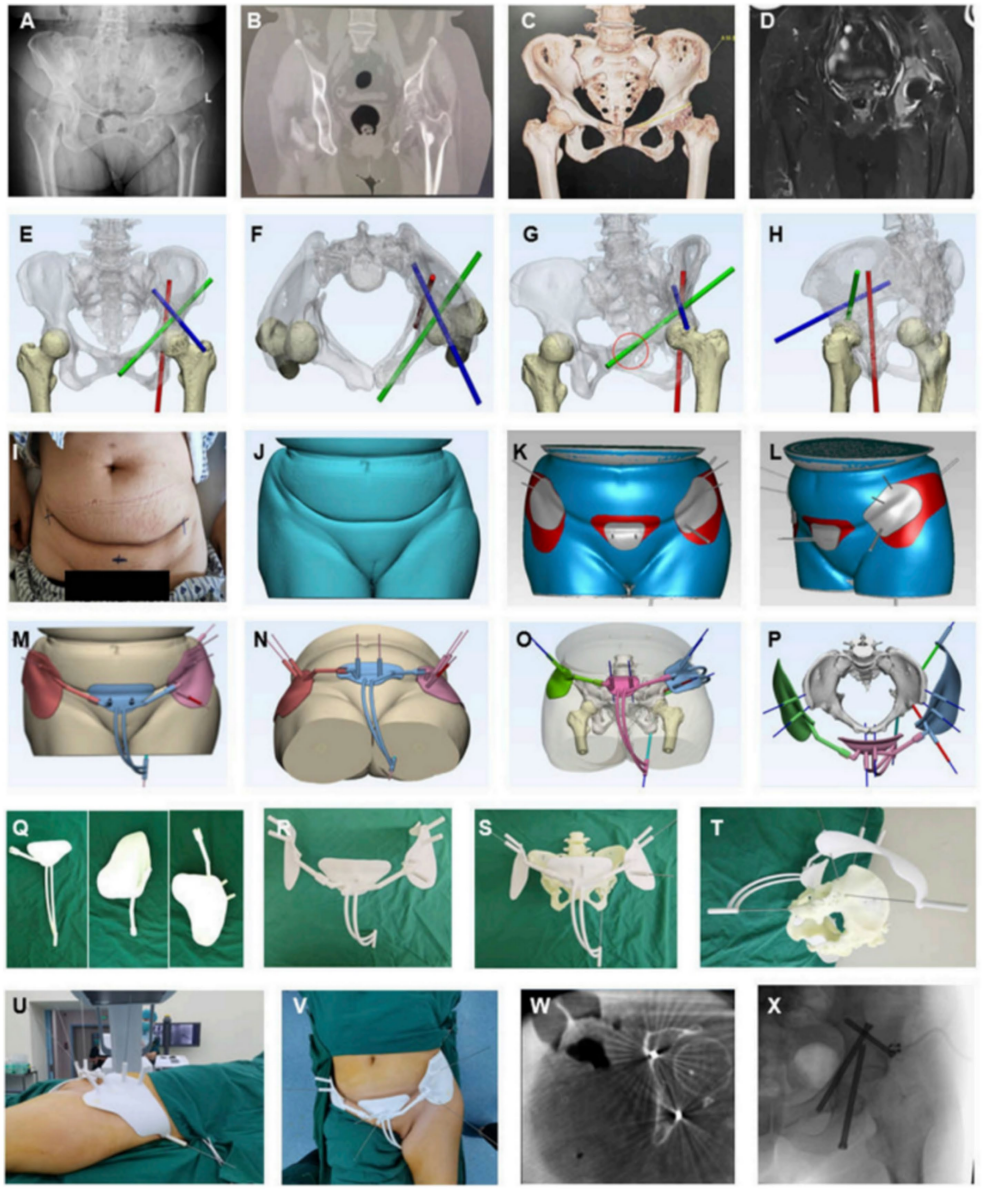
AI-assisted preoperative planning and 3D-printing guiding frame for percutaneous screw reconstruction in periacetabular metastatic cancer patients. **(A–D)** Preoperative imaging (X-ray, CT, 3D reconstruction, MRI). **(E–H)** Convolutional neural network–based determination of screw entry points and trajectories. **(I–L)** Skin surface recognition algorithm for personalized anchoring pads. **(M–P)** Guiding frame generation with trajectory tubes and anchoring feet. **(Q–T)** 3D-printed components and assembled guiding frame. **(U–X)** Intraoperative application, CT and fluoroscopic validation, and postoperative screw fixation. Reproduced under terms of the CC-BY license (77). Copyright 2024, Frontiers in Bioengineering and Biotechnology.

In preoperative modelling, AI markedly improves the automation and accuracy of three-dimensional reconstruction. By integrating CT and MRI with deep learning segmentation networks, AI swiftly delineates skeletal, soft tissue, and lesion boundaries, thereby generating patient-specific 3D anatomical models (74). For example, Lu et al. (75) developed an AI-assisted system that enabled rapid modelling and force-line assessment in complex pelvic fractures. This system substantially reduced modelling time while maintaining anatomical consistency scores comparable to manual modelling. Moreover, such systems automatically calculate key intraoperative parameters (e.g., osteotomy angles, implant trajectories), thereby providing quantitative support for optimising preoperative plans (76) (Figure 7).

Notably, AI-enabled intraoperative navigation systems increasingly achieve real-time fusion of multimodal images. Conventional systems primarily rely on CT registration but suffer from accuracy limitations caused by soft tissue occlusion and bone displacement. By integrating intraoperative fluoroscopy, preoperative CT/MRI, and optical tracking, multimodal AI navigation constructs dynamically updated surgical views. In particular, Zhang et al. (77) developed an AI navigation platform that achieved submillimetre accuracy in pedicle screw placement. The system aligns intraoperative C-arm images with preoperative 3D models and corrects patient drift in real time, thereby reducing penetration failure rates significantly.

Additionally, augmented reality (AR) and virtual reality (VR) (78), enhanced by AI, are promoting seamless integration between preoperative planning and intraoperative execution. Multimodal inputs—including skeletal CT, vascular MRI, and functional imaging—create interactive virtual environments that support preoperative rehearsal and pathway simulation. During surgery, AR devices overlay AI-generated surgical plans onto the patient’s body in real time, thereby delivering navigation consistent with physical reality. Research shows that AR-assisted total knee arthroplasty outperforms conventional methods in osteotomy precision, prosthesis alignment, and operative time, thereby highlighting its clinical potential (79, 80).

In summary, multimodal AI has established an integrated workflow spanning static modelling to dynamic navigation for preoperative planning and intraoperative guidance. Ultimately, this advancement enhances surgical precision and safety and lays the groundwork for future personalised orthopaedic surgery.

### 4.6 Efficacy prediction and intelligent treatment decision-making

Within precision medicine, efficacy prediction and personalised treatment are becoming increasingly central to orthopaedic clinical decision-making. Conventional efficacy assessments still depend on postoperative imaging and subjective scoring, yet they lack feedback mechanisms to optimise preoperative interventions. The emergence of multimodal AI now enables efficacy prediction by integrating imaging, clinical, and bioinformatics data, thereby facilitating both the formulation and dynamic adjustment of personalised treatment strategies.

AI constructs predictive models by integrating diverse preoperative factors, including imaging data, biochemical markers, and electronic medical records. This approach enables quantitative assessment of postoperative recovery outcomes (81). For example, in hip replacement, Zhang et al. (82) developed a multimodal predictive model that integrates CT/MRI radiomic features with clinical indicators such as demographics and preoperative pain scores. This model accurately predicted postoperative functional improvement (e.g., Harris and WOMAC scores) and demonstrated markedly superior performance compared with single-modality models. Consequently, such preoperative risk–benefit assessment provides valuable data to support individualised surgical planning, recovery timeline estimation, and postoperative management.

Furthermore, AI contributes to postoperative rehabilitation monitoring and supports dynamic adjustment. By integrating postoperative X-rays, MRI, serum inflammatory markers, and gait data, AI models can detect deviations from rehabilitation trajectories, thereby issuing timely alerts for functional impairment or prosthetic malalignment (83). Notably, Lee et al. (84) demonstrated that AI-based camera gait analysis generates quantitative metrics strongly correlated with Harris hip scores, thereby enabling reliable prediction of postoperative recovery. Concurrently, Wang et al. (85) developed an AI framework that combines CT registration with personalised dynamic modelling, improving hip kinematic measurement accuracy and supporting personalised rehabilitation timeline prediction.

**Figure 8:**
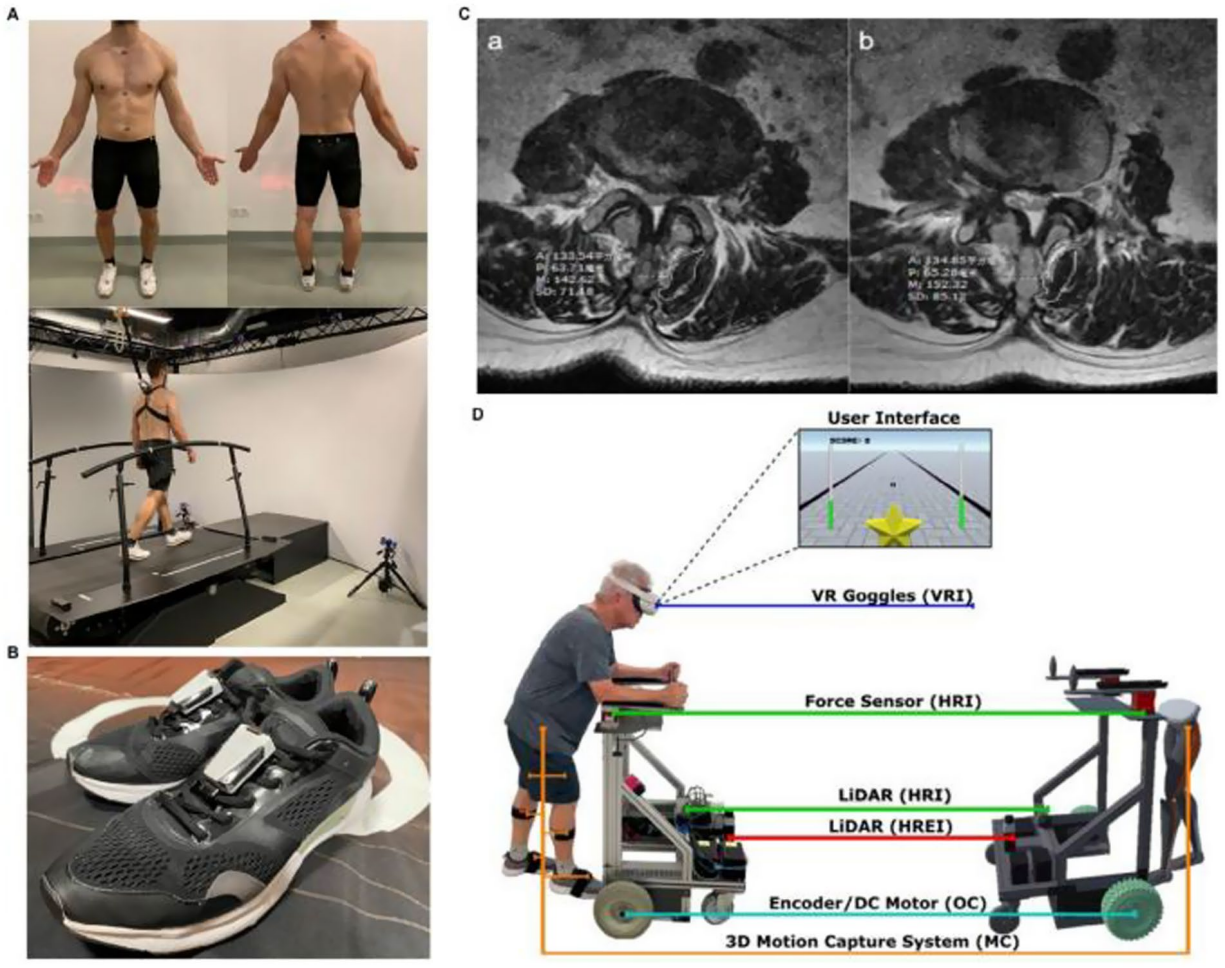
Applications of U-Net-Based Deep Learning Hybrid Model. **(A)** Gait analysis dataset generation using self-selected speed treadmill walking for 3D kinematic and dynamic assessment. **(B)** Wearable six-axis inertial sensors attached to shoes for stride speed and bilateral gait parameter collection. **(C)** Lumbar T2-weighted MRI analysis for postoperative rehabilitation monitoring, highlighting multifidus muscle cross-sectional area and related parameters. **(D)** Immersive virtual reality rehabilitation robot integrating VR goggles, force sensors, LiDAR, 3D motion capture, and DC motors for personalized training and real-time feedback. Reproduced under terms of the CC-BY license (91). Copyright 2025, MedComm.

Multimodal AI is widely applied in combined radiomics and molecular analyses to assess treatment sensitivity and drug resistance. For example, to predict the efficacy of biologic therapies for rheumatoid arthritis, researchers combined ultrasound data with haematological markers and transcriptomic/multi-omics features in machine learning models. This approach successfully distinguished responders from non-responders, demonstrating high discriminatory power in both prospective and retrospective cohorts (86, 87). It has also shown promise for assessing chemotherapy response in bone tumours and guiding personalised management of bone infections. Radiomics and radiogenomics models support preoperative efficacy evaluation, while AI integration into diagnostic and therapeutic workflows for bone and joint infections (7, 88, 89) may ultimately advance “treatment on demand.” Furthermore, AI aids personalised implant design and surgical pathway planning. By considering patient bone density, alignment parameters, and anatomical structures, AI generates customised implant geometries and performs virtual placement and biomechanical simulations. In addition, when integrated with navigation or robotic systems, AI further enhances intraoperative precision. Moreover, AI enables dynamic adjustment of rehabilitation protocols through closed-loop systems that incorporate wearable or “smart” implants and remote monitoring (90–92) (Figure 8).

In conclusion, multimodal AI is transforming traditional “experience-driven” treatment into “data-driven, mechanism-oriented, and individualised” approaches. Its integration into preoperative efficacy prediction, intraoperative pathway optimisation, and postoperative rehabilitation tracking provides the foundation for truly personalised orthopaedic treatment.

## 5 Challenges and perspectives

Despite notable progress in applying multimodal imaging and AI to orthopaedic diagnosis and treatment, widespread clinical adoption continues to face substantial challenges. Future research should therefore prioritise algorithm optimisation, data sharing, model generalisation, and the clinical translation of intelligent orthopaedic systems.

### 5.1 Data heterogeneity and standardisation challenges

Data heterogeneity is a well-known challenge in the development of AI models, particularly when working with multimodal imaging data from diverse sources. Variability in imaging modalities (e.g., X-ray, CT, MRI, PET), data acquisition protocols (e.g., scanner settings, contrast agents, image resolution), and patient demographics (e.g., age, sex, comorbidities) can create significant biases and inconsistencies in the training and validation datasets (93). These factors can affect the performance of AI models, often leading to overfitting to specific data sets or institutions, reducing the model’s ability to generalize across different clinical environments. Additionally, the lack of data standardization—for example, inconsistent image preprocessing, annotation practices, and quality control measures—further complicates model development. Without uniform standards, AI models may struggle to maintain reliable accuracy when applied to new, unseen data. This issue also poses significant barriers to multicenter validation, where datasets from multiple hospitals or imaging centers must be aggregated to assess model performance across different settings. A model trained on data from a single center or imaging modality may fail to perform optimally when deployed in a real-world clinical setting with diverse sources of imaging data. These challenges have significant implications for clinical translation. Even though AI models may perform well in controlled research environments, the variability of real-world clinical data means that models may not achieve the same level of success when deployed in routine clinical practice. The lack of data standardization across institutions means that clinical adoption will require additional efforts for data harmonization and cross-site validation to ensure that AI models can effectively support decision-making in diverse settings.

### 5.2 Insufficient model generalisation and robustness

Current AI models frequently achieve high performance on specific datasets. However, in real-world clinical settings spanning diverse devices, institutions, and patient populations, accuracy frequently declines due to variation in image quality and case complexity (94). For example, fracture detection models perform well on data from top-tier hospitals but deteriorate significantly on external datasets (e.g., ankle fracture detection AUC decreased from 0.95 in internal validation to 0.86 in external testing), underscoring substantial out-of-domain generalisation challenges (95). To enhance generalisation, strategies such as transfer learning, multi-task learning, and few-shot learning are indispensable. Concurrently, incorporating active learning and reinforcement learning further enhances model adaptability to “unseen scenarios.”

### 5.3 Explainability and credibility issues

Notably, AI models in orthopaedic imaging face the “black box” challenge, as they cannot provide clinically acceptable interpretation pathways, thereby hindering adoption (96). Comprehensive diagnosis of orthopaedic diseases necessitates integration of clinical manifestations, imaging features, and intraoperative findings. Consequently, verifying both model explainability and credibility is essential (97, 98). Techniques including heatmaps, attention mechanisms, and visualisation modules can significantly enhance interpretability. Furthermore, interdisciplinary collaboration is crucial for establishing evaluation frameworks that ensure clinical pathway consistency.

### 5.4 Challenges in clinical integration and intelligent workflow embedding

The value of AI models is greatly diminished if they are not effectively embedded into clinical workflows or seamlessly integrated with PACS, preoperative planning platforms, or intraoperative navigation systems (99). In addition, the physician learning curve and entrenched work habits must be carefully considered when introducing AI systems (100). Consequently, future efforts should focus on refining interface design, interactivity, and real-time performance evaluation, while promoting end-to-end AI integration across workflows—from preoperative decision support and intraoperative navigation to postoperative outcome assessment.

### 5.5 Issues of regulatory ethics and privacy security

Medical AI must strictly adhere to ethical and regulatory frameworks, particularly when processing personal health information, to ensure anonymisation, privacy protection, and compliant data sharing (101). At present, most AI models have not undergone clinical registration, certification, or ethical review during development. This gap poses a major barrier to their translation into medical products (102, 103). Therefore, future work should strengthen regulatory guidance and support for AI compliance pathways, aiming to establish medical device registration systems and multicentre trial standards.

### 5.6 Future perspectives

Looking ahead, deeper integration of multimodal imaging and AI into orthopaedic care is expected to drive the development of new intelligent precision medicine models. Recent advances in lightweight neural networks and edge computing have catalysed a paradigm shift, enabling AI integration into mobile devices and intraoperative navigation platforms. This integration could transform healthcare by delivering real-time intelligent assistance at the clinical frontline. Concurrently, task-specific models for fractures, bone tumours, and joint degeneration will further enhance AI specialisation and clinical interpretability. Ultimately, intelligent orthopaedics is expected to evolve from single-purpose diagnostic tools to comprehensive Clinical Decision Support Systems. A defining feature of these systems is their integration of multi-source heterogeneous data, including imaging, pathology, physiological signals, and electronic health records. Such integration will enable early disease detection, dynamic monitoring, and personalised treatment recommendations. In the longer term, digital twins, disease progression modelling, and advanced visualisation platforms may enable the creation of “virtual skeletal individuals.” These could support personalised preoperative planning, predictive analytics, and long-term efficacy assessment. Achieving this goal will require interdisciplinary collaboration and large-scale sharing and integration of real-world data across multiple centres. At the same time, establishing clinical translation standards and robust ethical-regulatory frameworks is essential to ensure safe, effective, and continuously evolving AI applications in orthopaedic diagnosis and treatment.

## 6 Conclusion

The convergence of multimodal imaging and artificial intelligence is reshaping orthopaedic diagnosis and treatment, shifting from experience-driven practice to intelligent, data-driven precision medicine. Evidence indicates that multimodal AI enhances fracture detection, tumour characterisation, osteoarthritis evaluation, osteoporosis risk prediction, and surgical planning, underscoring its broad clinical potential. Nevertheless, challenges remain in data integration, model robustness, interpretability, and clinical translation. Future systems are expected not only to automate lesion detection and structural measurement but also to support treatment pathway decision-making, potentially evolving into AI-assisted decision-support tools for orthopaedic surgeons. To achieve this transformation, advances in standardised databases, self-supervised learning, model fairness, and real-world validation frameworks will be essential. Ultimately, multimodal AI holds immense promise to transition from laboratory validation to routine clinical application, enabling safer, more efficient, and more precise solutions for orthopaedic patients.
